# Predictors of undernutrition among the elderly in Sodo zuriya district Wolaita zone, Ethiopia

**DOI:** 10.1186/s40795-019-0320-9

**Published:** 2019-11-08

**Authors:** Kidest Wondiye, Netsanet Abera Asseffa, Tsegaye Demisse Gemebo, Feleke Hailemichael Astawesegn

**Affiliations:** 1 Terepeza Development Association (TDA), Wolaita District Office Project Facilitator, Wolaita sodo, Ethiopia; 2 School of Public Health, College of Health Sciences and Medicine, Hawassa University, Hawassa, Ethiopia; 3 School of Public Health, College of Health Sciences and Medicine, Wolaita Sodo University, Wolaita sodo, Ethiopia

**Keywords:** Elderly, Undernutrition, Associated factors, Southern Ethiopia

## Abstract

**Background:**

In any society, the elderly are among the vulnerable and high risk groups with regard to health status. In persons over the age of 60 years, nutrition is among the important determinants of health. However, undernutrition among the elderly is often under diagnosed and/or neglected. Hence, in this study, we looked at prevalence and factors associated with undernutrition among the elderly.

**Methods:**

A community based cross-sectional study was conducted at Sodo Zuriya district. Multi-stage systematic sampling method was used to select 578 elderly. A structured questionnaire was used to collect data on socio-demographics, dietary diversity, and health status of the elderly.

Measurements of weight and height were taken using digital weighing scale and stadio-meter, respectively. Data was entered and cleaned in Epi-Data version3.1and exported to SPSS version 20 for analysis. Binary and multivariate logistic regressions were done and odds ratios with 95% confidence intervals were calculated.

**Results:**

The overall prevalence of undernutrition was 17.1%. On multivariate logistic regression, being unable to read and write (AOR = 2.09), not being married (AOR = 2.02), history of decline in food intake (AOR = 2.1), smoking (AOR = 4.9) and monthly income <$20 (AOR = 7.5) were factors positively associated with undernutrition.

**Conclusion:**

The study revealed that prevalence of undernutrition in the district was relatively high. Hence, it is among the major public health burdens in the district. Hence, to improve nutritional status of elderly the district health office and health professionals should consider behavioral support interventions to assist in cessation of smoking. There is also a need to financially empower the elderly in the district.

## Background

Aging is an irreversible biological process which starts from conception and ends after death [1].. The world has seen substantial growth in the number of the elderly population and expected to become the largest demographic group in many countries in the next few decades*.* According to the United Nations, in 2025, it is estimated that the number of aged people 60 years or older will be 1.2 billion and 2 billion in 2050 representing about 22.0% of the world’s population [2]. Eleven percent of the world population and 5.0% of Ethiopian population were categorized under elderly population- aged ≥60 years [3, 4]. The rising life expectancy within the older population responsible for their number and proportion at old ages [5].

Malnutrition is the state of being poorly nourished which can be caused by excess (overnutrition) or lack of nutrients (undernutrition). In the aging group undernutrition is an important problem that has been seen in hospitals, residential care and in the community [6]. The prevalence of undernutrition is increasing among the elderly and associated with a decline in functional status, impaired muscle function, decreased bone mass, immune dysfunction, anemia, reduced cognitive function, poor wound healing, delayed recovery from surgery, higher hospital readmission rates, severe, morbidity and mortality [7].

Studies determining the prevalence of undernutrition among elderly shows prevalence ranges from 0 to 24.0% [8–10]. The overall prevalence of under nutrition among older people in sub-Saharan Africa is reported to be between 6 % in Cameroon, and 48.0% in Ghana. Older age group were more affected by malnutrition than their counter parts [11, 12, 14–16].

Undernutrition among elderly people is becoming significantly high regardless of the better progress on health care system. In a study done in Northwest Ethiopia, the prevalence of under nutrition among elderly people was 21.9% [3]. In Ethiopia, little attention has been given to the elderly despite their increasing number and nutritional needs. However, there are limited studies which assess the determinants of under nutrition in elderly people in southern Ethiopia. Therefore, the aim of this study was to determine the prevalence of undernutrition and to identify risk factors associated with undernutrition among elderly.

## Methods

### Study design and area

A community based cross sectional study design was employed in Sodo Zuriya district from February to June, 2017. Sodo Zuriya district was one of the districts in Wolaita Zone, Southern Ethiopia. It is located at 327Kms (Kilometers) far from Addis Ababa, capital city of Ethiopia and 160Kms from Hawassa, the regional capital. Based on the last census, it had a total population of 162,691, of whom 80,002 were men and 82,689 women. The number of older persons — those aged 60 years or over were 16,233. The district had 36 *Kebeles* (the smallest administrative unit in Ethiopia) [12]. The major type of cereal grow in the district was maize. The population mostly consumes root and tuber based products like potato, sweet potato, *godere (a greenish-purple potato)*, and cassava. Among fruits avocado, papaya, banana and mango were commonly consumed. From vegetables kale and cabbage were frequently used.

### Study population

Study population were elderly who were systematically selected and living in selected *Kebeles* of the Sodo Zuriya district. Those who were seriously ill and who cannot stand without aid were excluded from the study. Five participants were excluded from the study.

### Sample size determination and sampling technique

The sample size was calculated using a single population proportion formula,

$$ n=\frac{z_{1-\alpha /2}^2p\left(1-p\right)}{d^2} $$

The assumptions were, estimated prevalence for undernutrition of elderly, 21.9% from study done in Gonder town [3], margin of error 5.0%, design effect of 2 and 10.0% non-response rate. Accordingly, the final sample size became 578. A multi stage simple random sampling technique were applied to select ten kebeles out of 36 kebeles.. In the selected 10 kebeles there were 207 sub-*Kebeles*. Then we considered a proportional allocation to the sample size to allocate participant for each sub-*Kebeles*, finally systematic sampling technique was used to select households included in the study. The sampling interval was calculated by dividing the total households in the selected sub-*Kebeles* of the *Kebele* by the final sample size, which gave every third household. Then after, the first household was selected from each sub-*Kebeles*, by spinning a pen, where the tip of the pen was pointed taken to be the first household. Then the sampling interval was added on to the first household to identify the consecutive households.

### Data collection tool

The questionnaires contains socio-demographic factors, life style factors, 24 h food diary and nutritional factors, physiological factors, psychological factors and health related factors. During the preparation of the questionnaire, related published articles and Mini Nutritional Assessment Questionnaire were reviewed and contextualized. It was prepared in English and translated into Amharic and back translated into English to maintain consistency.

### Data quality assurance

Before actual data collection pre-test was done among 10% of sample size in relatively similar district but out of actual study area to check accuracy, to estimate time and any inconsistency and necessary corrections were made. Experts with a nutrition and dietetic background assessed content validity. Trained ten diploma holders in clinical nursing collected data and two bachelor holders supervised the collection. The data collection tool had two parts; interview using questionnaire and target population assessment using measurement scales. Regarding the target population assessment, weight and height were measured using digital weighing scale and Stadio-meter respectively. During the measurement participants were barefoot, legs straight, shoulders relaxed and look straight ahead at the horizontal plane. In addition, they were asked to inhale deeply, hold the breath and maintain an erect position just before taking the measurement. Reading of height measurement was taken twice to the nearest 0.1 cm. A digital weighing scale was used to measure weight; it was measured twice in light clothes without shoes and stand still in the middle of the scale’s platform; and the average weight was taken. The district grows most of the stable crops through out the year. Hence, the difference might not exist when it comes to when data was collected.

### Operational definition

#### Seriously ill

Those who were unable to communicate due to sickness were considered as seriously sick. Moreover, this study involved height measurement that requires being on barefoot, legs straight; shoulders relaxed and maintain an erect position. Those who were unable to do so were also excluded.

**Elderly:** a person whose age greater than 60 years [13].

#### Undernutrition

Elderly who had BMI less than 18.5 kg/m^2^. Those who had Body Mass Index (BMI) between 18.5 Kg/m^2^ to 24.9 Kg/m^2^ were considered as normal and between 25 and 29.9 Kg/m^2^ were considered as overweight and obese and coded as No whereas those with BMI of less than 18.5 Kg/m^2^ considered as undernutrition and coded as Yes [14].

The food groups’ classification were determined using the Food and Nutrition Technical Assistance (FANTA) description as Low Dietary Diversity Score (DDS): when an elderly person consume < 3 food items per day; Moderate Dietary Diversity Score (DDS): when an elderly consume 4–5 food items per day; High Dietary Diversity Score (DDS): when an elderly consume > 5 food items per day [15].

#### Malnutrition

We classified malnutrition using Subjective Global Assessment form (SGA). Which is validated tool to classify malnutrition clinically. Accordingly Mild under nutrition: no decrease in food/nutrient intake; < 5% weight loss; no/minimal symptoms affecting food intake; no deficit in function; Moderately malnourished: definite decrease in food/nutrient intake; 5–10% weight loss without stabilization or gain; mild/some symptoms affecting food intake; Severely malnourished: severe deficit in food/nutrient intake; > 10% weight loss which is ongoing; significant symptoms affecting food/ nutrient intake; severe functional deficit.

**Cigarette smoking:** was operationalized as subjects who smoked at least one cigarette per day at the time of the study was classified as current smokers and those who smoked for at least three years in the past but had stopped by the time of the study was classified as a habitual smokers. Got was defined as a block or village that contains minimum thirty households.

### Method of analysis

Data were entered into Epi-Data version 3.1 and exported to SPSS version 20 for analysis. All continuous data were checked for normality using histogram and other normal plots. Univariate analysis was used to determine the frequencies. Bivariate analysis was applied to determine associations between outcome and exposure variables. Independent variables having *P*-value less than 0.05 on bivariate analysis were candidates for multivariable analysis for further confounding effect control. Hosmer and Lemeshew goodness of fit test was done to assess the fitness of the model during multivariate analysis. With *p*-value of 0.25, the model was ensured being fit well for the multivariate analysis and accepted. Both crude and adjusted odds ratio with 95% confidence interval were reported to measure the strength of association between exposure and outcome variable. The results on multiple logistic regression were considered statistically significant at *P*-value< 0.05.

## Results

### Socio demographic characteristics of participants

Of the total 578, 554 elderly participated in the study giving response rate of 96.0%. The median age of participants was 65 years with (SD**+** 8.1). Among the study participants majority of them were aged 60–65 years, 52.2% (*n* = 281). Majority of the participants, 76.5% (*n* = 424) cannot read and write. Most of the participants, 83.0% (*n* = 460) had monthly income less than $20 (Table 1).

**Table 1:** Socio-demographic characteristics of elderly in Sodo Zuriya districts, Wolaita zone, southern Ethiopia 2017 (*n* = 554)

Variable	Category	N	%
Sex	Male	295	53.2
	Female	259	46.8
Age group	60–65	281	52.2
	66–79	195	35.2
	> 80	70	12.6
Religion	Orthodox	274	49.5
	Protestant	255	46
	Catholic	15	2.7
	Muslim	6	1.1
	Apostolic	4	0.7
Education status	Unable to read and write	424	76.5
	Primary	99	17.9
	Secondary and above	31	5.6

### Health and life style characteristics of participants

Regarding cigarette smoking and alcohol intake, 79.4% (*n* = 31) smoke on daily basis and 27.5% (*n* = 27) took alcohol on daily basis. Regarding health status of the participants, 49.3% (*n* = 273) were sick in the last three months. From the total participants, 33.6% (*n* = 183) of them were suffering from decline in food intake in the last three months and most mentioned loss of appetite as a reason. Of the total participants, 81.8% (*n* = 453) of participants do not feel lonely (Table 2).

**Table 2:** Health and Life style characteristics of elderly in Sodo Zuriya districts, Wolaita zone, southern Ethiopia 2017 (*n* = 554)

Variable	Category	n	%
Currently smoking	Yes	39	7.0
	No	515	93.0
Smoke daily(*n* = 39)	Yes	31	79.4
	No	8	20.6
Drink alcohol currently	Yes	98	17.7
	No	456	82.3
Frequency of alcohol use	Daily	27	27.5
	5–6 days per week	22	22.4
	1–4 days per week	16	16.3
	1–3 days per month	25	25.5
	Once per month	8	8.2
Illness in the last three months	Yes	273	49.3
	No	281	50.7
Frequent disease occurred	Joint pain	167	30.1
	Hypertension	40	7.2
	Diabetes mellitus	19	3.4
	Others^a^	47	8.5
Decline in food intake	Yes	183	33.0
	No	371	67.0
Reason for decline	Digestive problem	51	27.7
	Loss of appetite	86	47.0
	Chewing problem	28	15.3
	Loss of smell and taste	18	9.8
Take medication currently	Yes	118	21.3
	No	436	78.8
Reason for loneliness	Loss of family member	56	10.1
	Social isolation	27	4.9
	Other^b^	19	3.4

^a^ Acute febrile illness, skin diseases, ^b^ loss of assets

### Food frequency and dietary diversity characteristics

The most commonly consumed food groups in the last 24 h were dark green vegetables (81.9%), followed by dairy products (60.6%) and legumes and nuts (54.5%). Regarding the dietary diversity score (DDS), 57.0, 28.0 and 15.0% scored low, moderate and high respectively (Table 3).

**Table 3:** Consumption of the nine food groups by the study subjects in the last 24 h at Sodo Zuriya districts, Wolaita zone, southern Ethiopia 2017 (*n* = 554)

Variable	Categories	n	%
Any cereal and root	Yes	234	42.2
	No	320	57.8
Any dark green vegetables	Yes	454	81.9
	No	100	18.1
Other vitamin A rich fruits and vegetables	Yes	207	37.4
	No	347	62.6
Other fruits and vegetables	Yes	259	46.8
	No	295	53.2
Meat or fish	Yes	232	41.9
	No	322	58.1
Organ meat	Yes	221	39.9
	No	333	60.1
Legumes and nuts	Yes	302	54.5
	No	252	45.5
Milk and milk products	Yes	336	60.6
	No	218	39.4
Egg	Yes	83	15.0
	No	471	85.0
Dietary diversity score	High	83	15.0
	Moderate	155	28.0
	Low	316	57.0

### Prevalence of undernutrition

The overall prevalence of undernutrition among elderly in Sodo Zuriya districts was 17.1% while majority 80.6% (*n* = 446) have normal BMI and only 2.3% (*n* = 13) of them were overweight. Among the undernourished subjects 11.0% (*n* = 61) of them fall under mild category, 5.0% (*n* = 27) at moderate and 1.1% (*n* = 7) of them fall under severe category. The prevalence of undernutrition was slightly high among females 20.5% compared to male 14.2%. The prevalence is also relatively high among study participants whose age are greater than 65 years 24.6% than those whose age are between 60 and 65 years 14.8%.

### Independent predictors of undernutrition among the elderly

On bivariate logistic regression, factors such as age group, being unable to read and write status, sex, not being married, illness in the last three month, weight lost, taking medication, decline in food intake, smoking and monthly income <$20 were positively associated with undernutrition. Whereas, on the multivariable logistic regression, being unable to read and write, not being married, decline in food intake, monthly income <$20 and smoking were positively associated with undernutrition (Table 4).

**Table 4:** Bivariate and multivariate logistic regression of factors associated with undernutrition among elderly at Sodo Zuriya districts, Wolaita Zone, southern Ethiopia, 2017 (*n* = 554)

Variable	Category	Undernutrition		95% C. I for OR	
		Yes (%)	No (%)	Bivariate (COR)	Multivariate (AOR)
Age group	60–79	62 (14.8)	358 (85.2)	1	
	> 80	33 (24.6)	101 (75.4)	1.6 [0.33–0.83]**	0.7 (0.42–1.25)
Read & Write	Unable	84 (19.8)	340 (80.2)	2.3 [1.37–5.21]**	.2.09 (1.02–4.27)***
	Able	11 (8.5)	119 (91.5)	1	
Smoke currently	Yes	7 (43.8)	9 (56.2)	3.9 [1.43–10.92]**	4.9 (1.53–15.45)***
	No	88 (16.4)	450 (83.6)	1	
Sex	Female	53 (20.5)	206 (79.5)	1.4 [0.4–12.07]**	0.9 (0.55–1.53)
	Male	42 (14.2)	253 (85.8)	1	
Illness in the last three month	Yes	57 (20.9)	216 (79.1)	1.5 [1.07–2.64]**	1.3 (0.67–2.56)
	No	38 (13.5)	243 (86.5)	1	
Weight lost in the last three month	Yes	30 (23.6)	97 (76.4)	1.5 [1.06–2.88]**	1.2 (0.53–2.12)
	No	65 (15.2)	362 (84.8)	1	
Marital status	Married	44 (12.3)	314 (87.7)	1	
	Others*	51 (26.0)	145 (74)	2.1 [0.25–0.66]**	2.02 (1.26–3.43) ***
Take medication currently	Yes	27 (22.9)	91 (77.1)	1.5 [0.92–2.65]**	1.2 (0.63–2.35)
	No	68 (15.6)	368 (84.4)	1	
Decline food intake	Yes	47 (25.7)	136 (74.3)	1.9 [1.48–3.66]**	2.1 (1.02–4.23)***
	No	48 (12.9)	323 (87.1)	1	
Monthly income ($)	< 20	92 (20.0)	368 (80.0)	7.5 [2.35–24.42]**	7.5 (2.12–26.56)***
	> 20	3 (3.2)	91 (96.8)	1	

*Divorced or Widowed, **p-value< 0.25, ***p value< 0.05

Elderly who cannot read and write were two times more likely to be undernourished than those who can read and write (AOR = 2.09, 95%CI: 1.02–4.2). Study participants whose marital status either widowed or divorced were two times more likely to be undernourished than those married (AOR = 2.02,95%CI: 1.2–3.4). This study also showed that elderly who have history of decline in food intake had 2.1 times odds of being undernourished than their counter parts (AOR = 2.1, 95%CI: 1.02–4.2). Those who smoke on daily basis were five times (AOR = 4.9, 95%CI: 1.5–15.4) more likely to be undernourished than those who do not. Elderly whose monthly income less than 500 ETB (Ethiopian Birr) ($20) was 7.5 times (AOR = 7.5, 95%CI: 2.1–26.5) high likely to be undernourished than their counterparts (Table 4).

## Discussion

The overall prevalence of undernutrition was 17.1%. The prevalence was high among females 20.5% compared to males 14.2%. This finding is comparable with the studies done in northwest Ethiopia where 21.9%, Ghana 18.0% and Derham city of India (21.8%) were undernutrition [1, 3]. And it was lower as compared to study done in Tamil Nadu of India (41.3%), Brazil (28.9%) and lake Victoria basin of Tanzania (26.4%) [16–18]. However, it was higher than that of the study conducted in southwest Nigeria 2.9% [23]. The difference could be due to variation in socio-economic status of study population and some of the studies focused on urban dwellers.

This study pointed out that 57.0% of the elderly had low dietary diversity score. This finding is higher than a cross sectional study done in Northwest Ethiopia [1]. This difference might be related to the fact that their study was conducted in urban settings where people are less affected by poverty and food insecurity.

In this study, being unable to read and write were 2.09 times (AOR = 2.09, 95%CI: 1.02–4.2) more likely to be undernourished than those who can read and write. This is similar in other reported studies; Bangladesh and elderly of Lebanese living in rural settings [3, 8, 19]. The reason might be that knowledge has contribution for ones individual to consume diversified food and educated people are more likely to have good feeding practice and better life style.

Being widowed or divorced were two times was found to be an independent predictor of undernutrition. The result was similar to those in previous reports in Ghana and Portugal [20, 21] where majority of the study participants were married and had normal BMI while widowed or single were found to be underweight. The similarity might be related to exposure the elderly social isolation, loneliness, depression, financial worries that will affect food intake as a result increase likelihood of being undernourished.

In our study, decreased food intake was positively associated with undernutrition as compared to their counterpart. This finding is in-line with reports from Finland and India which illustrated significant relationship between nutritional status and declining food intake [22–24]. This might be related to increased age which in turn reduce the natural drive to eat and drink and resulting in anorexia of aging. These age related changes are regarded as an adaption to the natural decrease in energy requirement but also predispose older people to malnutrition by increasing the risk of an extreme reduction in food intake.

Another positively associated factor to develop undernutrition was cigarette smoking. This shows that unhealthy life style affect nutritional status of elderly as supported by a study conducted in northern Ghana where use of tobacco or cigarette smoking had effect on lowering BMI of the elderly [20]. In addition, smoking compromises the need to take food as well as fruits and vegetable that are rich in vitamin c and carotene.

Moreover, monthly income of less than $20 had significant association with undernutrition; similar to studies done in India and France which showed low income has negative effect on nutrition status of elderly [23–25]. This proves that nutrition-related health is closely related to socioeconomic status. Limited money may mean older people choose foods they would prefer not to eat. Being unable to afford and inadequate food intake could lead to poor nutritional status in elderly [26].

The findings of this study are generalizable to the region’s population because the participants’ socio-economic, educational status and way of life is unbiased reflection of the southern region elderly population [27]. This study has its own limitations. Information obtained from elderly on their health status, socio-demographic background, life style and their dietary intake might have recall bias. Since this study used physical measurements, there was probability of measurement and instrument error; as age increase there will be progressive and generalized loss of skeletal muscle and decrease bone height and density which affects their BMI during physical measurement which might over estimated prevalence of undernutrition in this study. Moreover, not examining oral, dental factors and data whether they own land or not was collected were limitations.

## Conclusion

The overall prevalence of undernutrition among the elderly in the district was relatively high. Undernutrition is therefore an important public health burden among the elderly in the study area. Being unable to read and write, not being married, decline in food intake, monthly income <$20 and being smoker currently on daily basis were factors positively associated with undernutrition.

Hence, to improve nutritional status of elderly the district health office and health professionals should consider behavioral support interventions to assist in cessation of smoking, and socialization with others.. There is also a need to financially empower the elderly in the district. Further studies are needed to generate a database for effective policy making and formulate a national policy on the nutrition and health of the elderly targeting the whole population to ensure healthy aging.

## Data Availability

The dataset analysed for the findings of this study are available with the corresponding author and can be accessed by reasonable request.

## Abbreviations

AOR: Adjusted Odds Ratio
BMI: Body Mass Index
CI: Confidence Interval
COR: Crude Odds Ratio
DDS: Dietary Diversity Score
ETB: Ethiopian Birr
FANTA: Food and Nutrition Technical Assistance
